# Supplementary material for: Depletion of VGLL4 Causes Perinatal Lethality without Affecting Myocardial Development
Source: Cells. 2022 Sep 10;11(18):2832. doi: 10.3390/cells11182832 (PMC9496954; doi:10.3390/cells11182832)
Supplement: Supplementary file 1 [file cells-11-02832-s001.zip › cells-1855605-supplementary.pdf]

cells

MDPI

Article

Depletion of VGLL4 Causes Perinatal Lethality without Affecting Myocardial Development

Caroline Sheldon [1], Aaron Farley [1,2], Qing Ma [3], William T. Pu [3] and Zhiqiang Lin [1,*]

1 Masonic Medical Research Institute, 2150 Bleecker St., Utica, NY 13501, USA
2 Biology and Chemistry Department, SUNY Polytechnic Institute, 100 Seymour Rd., Utica, NY 13502, USA
3 Department of Cardiology, Boston Children's Hospital, 300 Longwood Ave, Boston, MA 02115, USA
* Correspondence: zlin@mmri.edu

Expanded material and experimental procedures

Crispr-sgRNA mediated germ line mutation

Cas9 mRNA and gRNA were injected into the pronuclei of fertilized embryos, which were then transplanted into pseudopregnant mice. Two guide RNAs were used to cause deletion in the coding region of *Vgll4* TDU domain: 5'-gaaattaatacgactcactatagGCTCCTTGTAGTTCTTGCCCgttttagagctagaaatagc-3' and 5'-gaaattaatacgactcactataggCCATGTGTCACCTAGTGCTTgttttagagctagaaatagc-3'. F0 offsprings were screened for mutations with PCR, and the mutant carrying the largest deletion (46 bp deletion) was crossed to wild type mice to generate F1 pups.

Vgll4 flox targeting vector construction

pCMV plasmid was used as a backbone to generate Vgll4 flox targeting vector. Briefly, the PCR products of the left and right recombination arms were cloned into pCMV with EcoRI+KpnI and SalI +NheI respectively, to generate pCMV-Vgll4-L-R. Floxed Vgll4 exon 5 coding sequence was then cloned into the pCMV-Vgll4-L-R vector using KpnI+SalI restriction enzyme set. At last, Neomycin resistant cassette flanked with FRT sequences was placed between the right side LoxP site and the right recombination arm. At 5 prime site of the right recombination arm, one A>G mutation was introduced to destroy guide RNA PAM sequence.

Primary antibody used in this study.

VGLL4 polyclonal antibody was generated by Custom Antibody Service (YenZym, LLC), and a peptide containing 14 residues (HFRRSLGKNYKEPE, identical between mouse and human) was used as antigen to immunize rabbit. The elicited antibody was affinity-purified against the same peptide used for immunization. TNNI3 antibody (Santa Cruz, sc-365446), GAPDH (Sigma-Aldrich, G8795), YAP antibody (Protein Tech, 13584-I-AP), TEAD1 antibody (BD, 610923) for Co-IP, TEAD1 antibody (CST, 12292S), for Western blot.

Adult cardiomyocytes isolation

Adult Ventricular myocytes were isolated from 6–8 week old mice following a previously described method [1]. For cardiomyocyte dimension measurements, adult cardiomyocytes were first fixed with 4% PFA and then imaged with Keyence BZ-X800 microscope. For protein and RNA isolation, adult cardiomyocytes were lysed with RIPA buffer and Trizol reagent, respectively.

Echocardiography (Echo) measurements.

Echocardiography measurements were performed with a VisualSonics Vevo 3100 ultrasound imaging system. For adult mice, before being tested, the mouse chest was depilated and the mice were trained for echo for three consecutive days. Briefly, the mice were held in a position to expose the chest, and an artificial plastic probe was put onto the chest to mimic the action of the ultrasound probe. On the third day, conscious mice were subjected to Echo measurements.

Histology and immunostaining

For cryosection, hearts were fixed in 4% PFA, cryoprotected with 30% sucrose, and embedded in OCT. For embryo serial section, E16.5 embryos were dehydrated and embedded in paraffin. Hematoxylin and eosin (H&E) staining was performed on paraffin-embedded sections. Immunostaining was performed on cryosections and detected with Alexa-labelled secondary antibodies. EdU was administered intraperitoneally at 200 μg/animal (pregnant dams), 2 hours before tissue collection. EdU was detected with Click-iT chemistry (Invitrogen). TNNI3 antibody was used to visualize cardiomyocytes. Imaging was performed on a Keyence BZ-X800 microscopy system or a Zeiss LSM-700 confocal microscope.

Reference

[1] O'Connell, T.D., Rodrigo, M.C. and Simpson, P.C. (2007) Isolation and culture of adult mouse cardiac myocytes. *Methods Mol Biol* **357**, 271-296.

Supplemental Table S1. Primers used in this study.

Primer Name	DNA Oligo Sequence (5'–3')
Pr-623	GGAATAAGACTGTCAACGGAGACT
Pr-624	TCCTACAGAAAATATAGGGGCCTG
Pr-900	AGCGTTGGCTACCCGTGATATTGCTGAAGA
Pr-901	CAGGTCGGTCTTGACAAAAAGAAC
Pr-705	TAGAGCACGTGTGCACCTTGAGTTGGC
Pr-706	CCGTGCTGTTCTGTTGCCACAC
Vgll4 qRT-PCR TDU1 Forward	ACTGCCACCTGTGACCCTGTGGT
Vgll4 qRT-PCR TDU1 Reverse	CTGCTTTGATCTGAAGCCATGTGTC
Vgll4 qRT-PCR 5 prime Forward	ATGAACAACAATATCGGCGTTCT
Vgll4 qRT-PCR 5 prime reverse	GGGCTCCATGCTGAATTTCC
CycB1 qRT-PCR Forward	AAGGTGCCTGTGTGTGAACC
CycB1 qRT-PCR Reverse	GTCAGCCCCATCATCTGCG
Cdk1 qRT-PCR Forward	TTTCGGCCTTGCCAGAGCGTT
Cdk1 qRT-PCR Reverse	GTGGAGTAGCGAGCCGAGCC
Igf1r qRT-PCR Forward	CTTTGCGAGAACCATGCCAG
Igf1r qRT-PCR Reverse	TAGACGGTTGAGTTTGGCCC
Ccn2 (Ctgf) qRT-PCR Forward	CAAGGACCGCACAGCAGTT
Ccn2 (Ctgf)qRT-PCR Reverse	AGAACAGGCGCTCCACTCTG
Ccn1(Cyr61) qRT-PCR Forward	GCTCAGTCAGAAGGCAGACC
Ccn1(Cyr61) qRT-PCR Reverse	GTTCTTGGGGACACAGAGGA
Birc 2 qRT-PCR Forward	ACTTCAGACACCCCAGGAGAAGAAA
Birc 2 qRT-PCR Reverse	CCGCTGAACCGTCTGTCTCACC
Vgll4 NM_177683.3 Forward	CCGAATAGAAGACACAATCAGACACC
Vgll4 NM_177683.3 reverse	CTCCTAAAACTTCAGGAAGACAGAGG
Vgll4x4 XM_006506014.4 Forward	TTGGCAATGATTAAAGTGAG
Vgll4x4 XM_006506014.4 Reverse	TGTGTCACTGCTGTTCTTAGTCAG
Vgll4x3 XM_006506013.5 Forward	GACAGACTCGAAGGGATTCTAGAATC
Vgll4x3 XM_006506013.5 Reverse	CTGTGGAGAGTGTCAATACAGCTATG
Vgll4x1 XM_006506010.3 Forward	CTCTGAGTGTTCTTATCCCAGACTG
Vgll4x1 XM_006506010.3 Reverse	GAGGGAAGCTTCACGTTTTCATC

Supplemental Table S2. Summary of *Vgll4* transcript variants.

Gene ID	Gene Symbol	Transcript	Length (nt)	Protein	Length (aa)	Protein name	Isoform	Organism
232334	Vgll4	XM_006506010.3	3324	XP_006506073.1	281	VGLL4	X1	Mus musculus
232334	Vgll4	XM_036166060.1	2979	XP_036021953.1	250	VGLL4	X2	Mus musculus
232334	Vgll4	XM_006506012.3	2972	XP_006506075.1	250	VGLL4	X2	Mus musculus
232334	Vgll4	XM_006506014.4	2869	XP_006506077.1	201	VGLL4	X4	Mus musculus
232334	Vgll4	XM_006506013.5	12628	XP_006506076.1	243	VGLL4	X3	Mus musculus
232334	Vgll4	NM_001356371.1	2739	NP_001343300.1	281	VGLL4	2	Mus musculus
232334	Vgll4	NM_177683.3	2824	NP_808351.1	287	VGLL4	1	Mus musculus

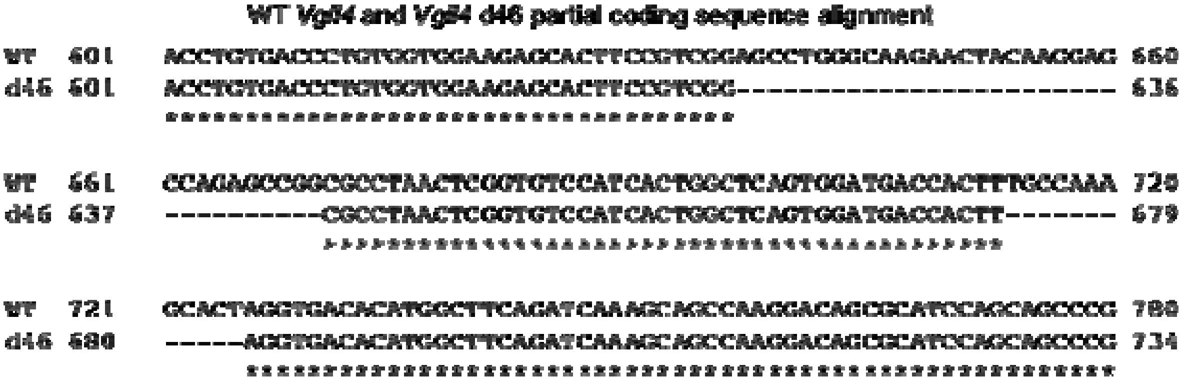

Supplemental Figure S1. DNA sequence alignment displaying *Vgll4* d46 indel mutation. DNA sequence alignment between wild type *Vgll4* (NM_177683.3) and *Vgll4* d46 allele. DNA sequence covering the mutated region was included in this alignment.

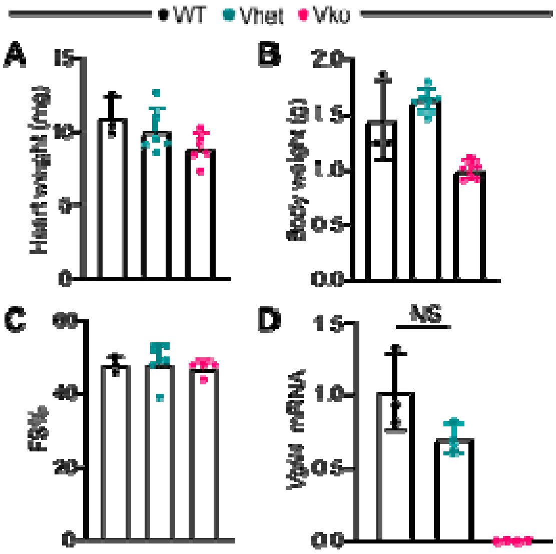

Supplemental Figure S2. *Vgll4* heterozygous mice were not distinguishable from wild type mice. *Vgll4* heterozygous mice were not distinguishable from wild type mice. **A**. Heart weight. **B**. Body weight. **C**. Fraction shortening. **D**. qRT-PCR measurement of *Vgll4* mRNA. Primers amplifying the TDU1 domain was used. A, B, C, and D, all data were collected from P0 or P1 pups.

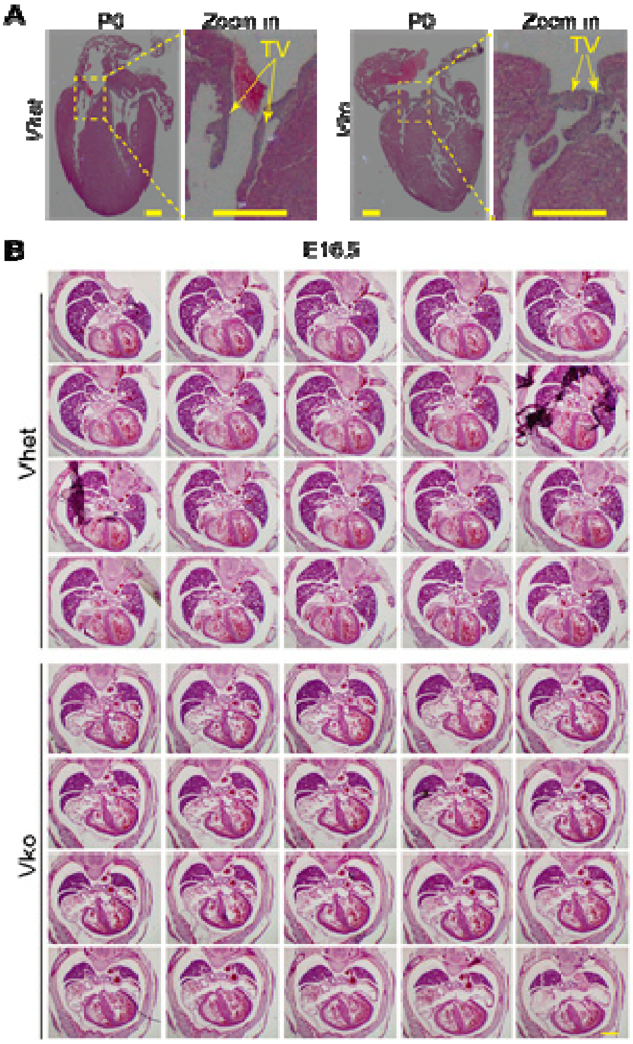

Supplemental Figure S3. Histology of the embryonic and neonatal *Vko* hearts. **A**. H&E stained P0 heart sections. Scale bar = 1 mm. TV, tricuspid valve. **B**. Serial section images of E16.5 hearts. Scale bar = 1 mm.

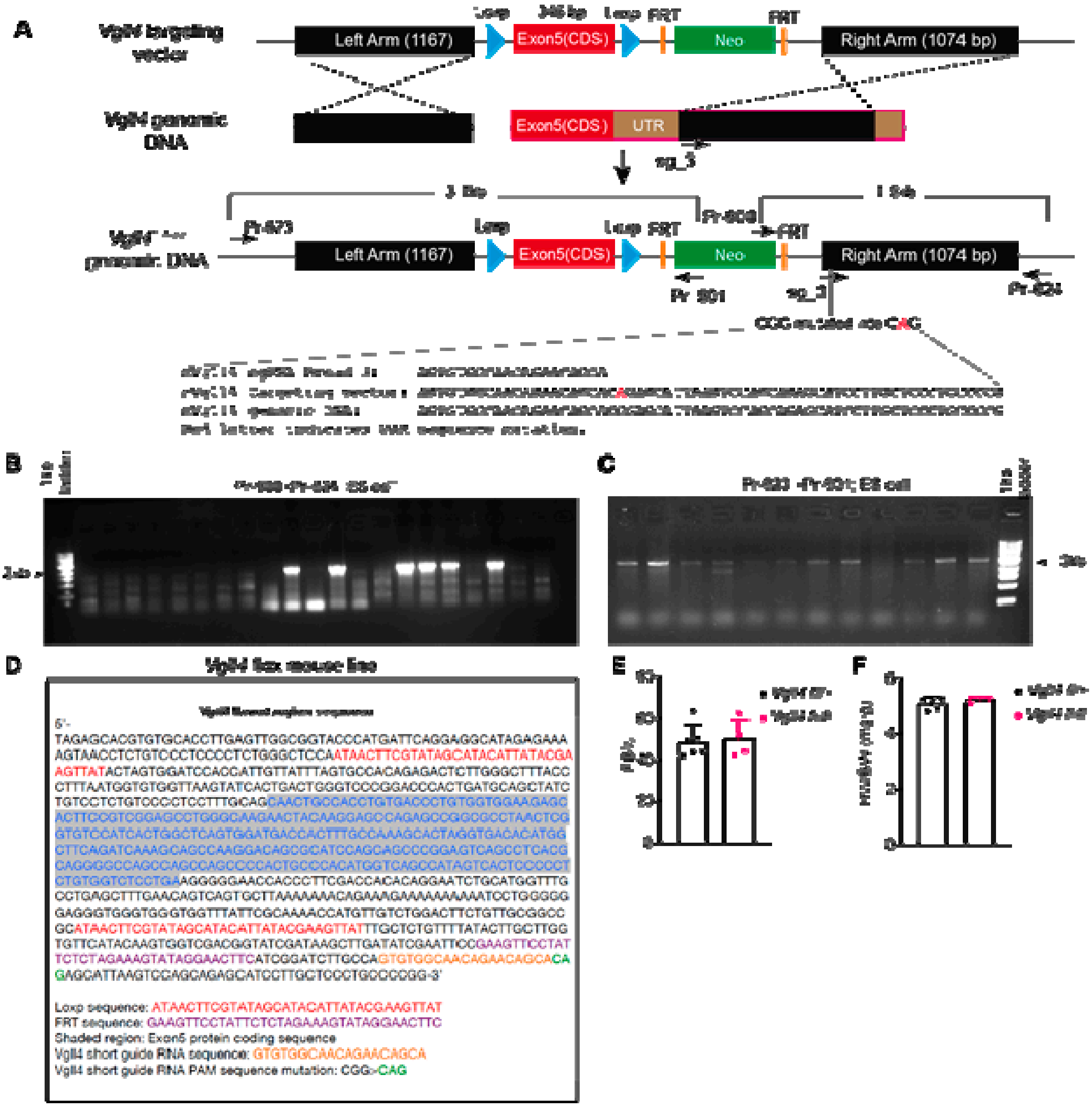

Supplemental Figure S4. Generation and validation of the*Vgll4* flox allele. **A**. *Vgll4* genome targeting strategy. The left and right recombination arms target *Vgll4* intron 4 and exon 5 untranslated region, respectively. A PGK::Neomycin selection cassette flanked by FRT sites was used for selection of recombinants, and *Vgll4* protein coding sequence (CDS) in exon 5 was flanked by LoxP sites. In the *Vgll4* flox genomic DNA diagram, the relative positions of primers and short guidance (sg) RNA sequence were labeled, and the PCR products sizes were indicated. Red letter indicates mutated PAM sequence. **B**,**C**. PCR screen of targeted mouse embryonic stem (ES) cells. The first round of PCR screen was performed with Pr-900+Pr-624 primer set (B), and the positive clones were further confirmed with pr-623+pr-901 PCR screen (C). **D**. Sequencing result of the *Vgll4* floxed region. Genomic DNA from *Vgll4*$^{fl/fl}$ mouse was sequenced to confirm the right insertion of LoxP sequences and the successful removal of Neomycin resistant cassette. **E**. Fraction shortening (FS%) and Heart to body weight ratio.

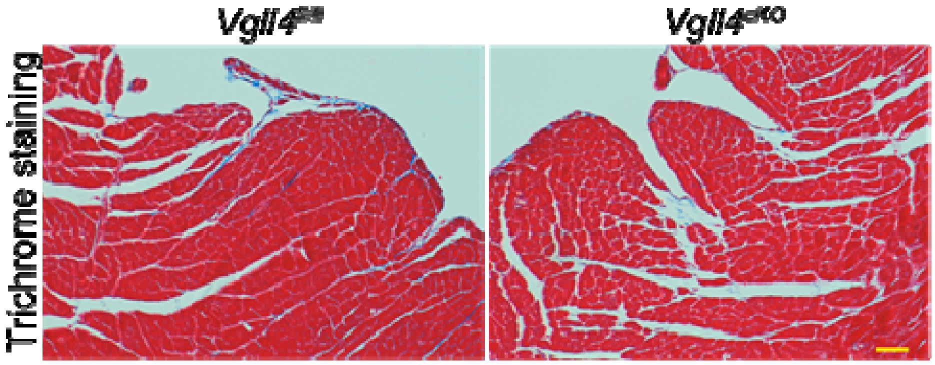

Supplemental Figure S5. Adult myocardium fibrosis analysis. Trichrome staining of adult myocardium. Scale bar = 50 µm.

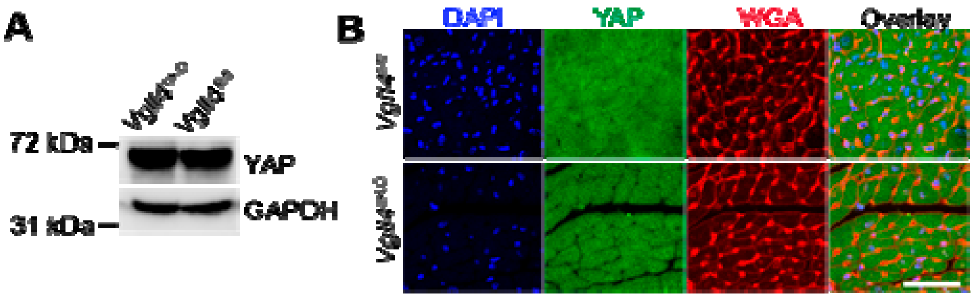

Supplemental Figure S6. Cardiomyocyte-specific depletion of VGLL4 does not change YAP expression and sub-cellular localization. **A**. YAP western blot. Total proteins from heart lysates were used for western blot. GAPDH was used as loading control. **B**. Immunofluorescence images of YAP antibody-stained adult myocardium. Wheat germ agglutinin (WGA) was used for visualizing plasma membrane. Scale bar = 50 µm.
